# Walking humans and running mice: perception and neural encoding of optic flow during self-motion

**DOI:** 10.1098/rstb.2021.0450

**Published:** 2023-01-30

**Authors:** Edward A. B. Horrocks, Isabelle Mareschal, Aman B. Saleem

**Affiliations:** ^1^ Institute of Behavioural Neuroscience, Department of Experimental Psychology, University College London, London WC1H 0AP, UK; ^2^ School of Biological and Behavioural Sciences, Queen Mary, University of London, London E1 4NS, UK

**Keywords:** optic flow, mouse vision, human vision‌, locomotion, psychophysics

## Abstract

Locomotion produces full-field optic flow that often dominates the visual motion inputs to an observer. The perception of optic flow is in turn important for animals to guide their heading and interact with moving objects. Understanding how locomotion influences optic flow processing and perception is therefore essential to understand how animals successfully interact with their environment. Here, we review research investigating how perception and neural encoding of optic flow are altered during self-motion, focusing on locomotion. Self-motion has been found to influence estimation and sensitivity for optic flow speed and direction. Nonvisual self-motion signals also increase compensation for self-driven optic flow when parsing the visual motion of moving objects. The integration of visual and nonvisual self-motion signals largely follows principles of Bayesian inference and can improve the precision and accuracy of self-motion perception. The calibration of visual and nonvisual self-motion signals is dynamic, reflecting the changing visuomotor contingencies across different environmental contexts. Throughout this review, we consider experimental research using humans, non-human primates and mice. We highlight experimental challenges and opportunities afforded by each of these species and draw parallels between experimental findings. These findings reveal a profound influence of locomotion on optic flow processing and perception across species.

This article is part of a discussion meeting issue ‘New approaches to 3D vision’.

## 1. Introduction

**Figure 1.**
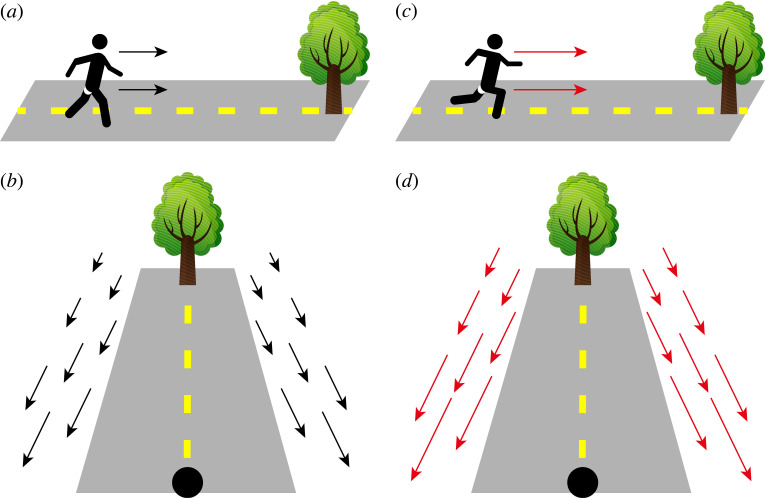
Introduction to optic flow during locomotion. When a subject is walking toward a tree (*a*), they experience a characteristic optic flow pattern (*b*) that is expanding outwards from the target location. When the subject moves faster (*c*) they experience a correspondingly faster optic flow (*d*).

Locomotion produces full-field optic flow that often dominates the visual motion inputs to an observer (figure 1) [1–7]. The perception of such visual motion is important for animals to guide their own movement within an environment and also to determine the relative movement of external objects, for example during prey capture [8,9] or predator avoidance [10]. Understanding how locomotion influences optic flow processing and perception is therefore essential to understand how animals successfully interact with their environment.

Perception and neural encoding of optic flow has historically been studied in stationary, fixating subjects. While these paradigms afforded tight control over experimental conditions necessary to investigate visual processing, they also have clear ethological limitations. Recent developments of experimental methods are however enabling a growing focus on the influence of self-motion and active behaviour on visual processing and perception [11–18]. In this article, we review research investigating the effects of self-motion on optic flow processing and perception, with a particular focus on movements relevant to locomotion. Throughout, we consider experimental research using humans, non-human primates and mice. In doing so we highlight the experimental challenges and opportunities afforded by each of these species, and, where possible, draw parallels between experimental findings obtained from each of them.

As the review is multidisciplinary, we first introduce some primers below to provide brief contextual background useful for reading this review.

### [Primer A] Perceptual functions of optic flow

Movement of an observer through an environment produces relative motion of the 3D environment. This relative environmental motion is focused through the optics of the eyes onto the retinas, forming two-dimensional velocity fields, i.e. optic flow, which are influenced by both self-motion and the structure of the environment [5,18,19]. Perceiving optic flow, therefore, enables an observer to make inferences about their movement within an environment. Indeed, perception of optic flow can be used to estimate the *direction* [20] and *speed* [21,22] of self-motion, gain information about the *structure and layout of objects within the environment* [23,24] and *parse external object motion* from visual motion due to self-motion (*flow parsing*; [25]).

### [Primer B] Visual cues for motion-in-depth and optic flow

A number of monocular and binocular visual cues provide information about optic flow and self-motion to an observer. Monocular cues include structured velocity fields as well as changes in the size and spatial frequency of visual objects [19,26]. Binocular cues include inter-ocular velocity differences (differences in velocity produced by a moving object projected onto two spatially separated eyes), and changes in binocular disparity [5,27]. Studies investigating perceptual sensitivity have found that the use of monocular and binocular cues for motion-in-depth varies substantially both across the visual field [28–30] and with viewing distance [31]. More generally, well-known errors in the perception of motion-in-depth can be explained by Bayesian inference of noisy sensory signals [32], suggesting that the influence of different visual cues on motion perception may depend on their reliability. Intriguingly, when humans are provided with feedback on the accuracy of their performance in a 3D motion perception task they can learn to leverage different visual cues [33,34]. The usage of different visual cues for the perception of optic flow is therefore likely to be flexible and context-dependent. An important consideration is therefore how display devices used in experiments may alter their use compared to natural viewing.

### [Primer C] Optics of the primate and rodent visual systems

In this article, we review studies from primates and rodents. There are many obvious differences in the optics of the rodent and primate visual systems which have implications for optic flow processing. Whereas primate eyes face forward, most rodents have sideways-facing eyes. While such lateral placement gives rodents a larger field of view [35,36], it comes at a cost—the region of visual space where the field of view of the two eyes overlaps, known as the binocular zone, is substantially smaller in mice (approx. 40° compared to approx. 120° in humans; [37,38]) resulting in a smaller region where binocular cues for depth perception are available. Nevertheless, mice can discriminate stereoscopic depths [39] and, similar to primates, have neurons that are binocular disparity-sensitive, throughout visual cortex [37,40,41], indicating that binocular cues for depth are used by the mouse visual system.

The mouse retina is suitable for processing optic flow despite its low-acuity. While the density of photoreceptors in mouse retina is actually similar to that of primates [42–45], the smaller size of the mouse eye coupled with a larger field of view means that the photoreceptor per unit area of the visual scene is smaller than in primates [46]. Moreover, the high acuity of primate vision is largely due to the concentration of approximately 99% of cone photoreceptors within the fovea, an area that takes up approximately 1% of the retina [47]. As such, mouse vision is believed to be similar to that of primate peripheral vision [42,46,48]. Given the importance of peripheral vision in the perception of optic flow [49–51] and the use of optic flow in human observers to perceive visual events when low visual acuity is simulated [52], the mouse provides an appropriate model species for investigating perception and neural encoding of optic flow.

### [Primer D] Neural encoding of optic flow

The neural encoding of optic flow is best understood in non-human primates. In particular, neurons in the dorsal Medial Superior Temporal (MSTd) area, a higher visual area within the dorsal stream of the primate visual system, have large receptive fields often covering both ipsi- and contralateral halves of the visual field, indicating their suitability for the encoding of full-field optic flow caused by self-motion [53]. Indeed, neurons in MSTd are selective for complex visual motion patterns such as expansions, contractions, rotations and spirals [54–57] that can arise from combinations of head and eye-movements (see [58] for a recent review). Moreover, tuning for these complex visual motion patterns can be invariant to the precise form of the stimulus used [59], as well as its location within the visual field [57].

Tuning for complex optic flow patterns, as observed in primate area MSTd [54–57], has not yet been identified in the mouse. However, neurons in a number of mouse visual cortical areas are selective for specific combinations of binocularly presented drifting gratings simulating forwards and backwards translations, as well as rotations [60], indicating that selectivity for optic flow patterns may be present. In particular, it was found that higher visual areas RL/A, followed by AM and PM, were enriched with neurons selective for translation or rotation compared to V1 [60]. Interestingly, mouse visual area RL is biased to represent the lower visual field [61,62], indicating that visual motion in the lower visual field may be important for signalling self-motion in the mouse, reflecting the proximity of mouse eyes to the ground plane. In accordance with this, neurons in the mouse visual cortex with receptive fields in the lower visual field tend to respond more strongly to coherent visual motion [63]. It will be important for future work comparing the neural encoding of optic flow in mice and primates to consider their distinct ecological niches.

It is unclear to what extent an analogous area to primate MST exists in the mouse visual system. More generally, a number of studies have sought to determine whether the mouse visual system has distinct processing streams analogous to the dorsal and ventral streams in primates [61,64–70], however, these studies have sometimes produced conflicting results. Given that both selectivity for coherent visual motion and tuning for visual speed are widespread in mouse visual areas [63,70,71], there may be a more distributed code for visual motion and optic flow in the mouse visual system.

### [Primer E] Modulation of visual processing during locomotion

Many recent studies have investigated the influence of locomotion on visual processing in the mouse, often leveraging the spontaneous locomotion exhibited by mice when head-fixed on a treadmill [72,73]. In just over a decade these studies have revealed effects of locomotion throughout the mouse visual system: from the outputs of the retina [74,75], to thalamic and midbrain nuclei [76–80] and a range of cortical areas [73,80–83]. While we provide an overview of the main findings below, other reviews provide a more detailed account of the effects of locomotion and the pathways supporting them (e.g. [17]).

The effects of locomotion on visual processing are diverse and vary between visual areas and cortical layers [71,76,77,79,80,84,85] as well as genetically, physiologically and functionally defined cell-types [73,78,86–90]. Modulation of visual responses during locomotion has been described at multiple spatial scales. At a cellular level: membrane potentials show bidirectional changes [86,91,92]; spontaneous and evoked firing rates are altered [73,85]; and visual response dynamics are less transient [71]. In terms of visual tuning properties: locomotion increases spatial integration [93]; is linked to additive and multiplicative tuning gain for visual features such as orientation, direction and spatial frequency [84,94]; and also altered tuning preferences for visual speed [81]. Furthermore, joint tuning for optic flow speed and self-motion signals correlated with running speed have been described in a range of mouse visual areas [77,80,95], indicating that integration of self-motion and visual motion signals is widespread in the mouse visual system. At the scale of neural populations: locomotion is associated with reductions in pairwise noise correlations [76,96]; changes in LFP power spectra [73,96]; altered functional connectivity between brain areas [97] and changes in the geometry and structure of latent population activity [71,98].

While these changes in neural activity observed during locomotion generally indicate enhanced encoding of visual inputs, perceptual studies are thus far limited and provide mixed results as to whether locomotion also improves visual perception [91,99,100], with changes in behavioural performance likely dependent on the specific task context [100].

Are equivalent changes to visual processing also present in primates? A recent preprint found that locomotion also modulates visual responses in head-restrained marmosets, a non-human primate [101]. By contrast to mouse V1, where firing rates tend to increase during locomotion, firing rates were more likely to decrease in marmosets and the magnitude of firing rate changes was overall weaker. Interestingly, the effects of locomotion in marmosets varied between neurons responding in the fovea and the periphery, with the latter more likely to increase firing rates during locomotion. More generally, the authors noted that changes in firing rates correlated with locomotion could be explained as a shared gain factor across the recorded population in both mice and marmosets, suggesting that similar principles may underlie modulation of visual systems by locomotion in mice and primates. Further comparative experimental work should provide insights into these principles.

### [Primer F] Experimental methods for investigating visual perception during movement

An increasing number of experimental methods are available to investigate the neural encoding and perception of optic flow by enabling the presentation of visual inputs to large areas of the visual field (table 1). These display environments can be coupled to subject self-motion (table 2) in various ways, enabling the investigation of optic flow processing and perception during subject movement.

**Table 1.** An overview of the visual environments available to investigate the neural encoding and perception of optic flow. The table also summarizes available methods to couple visual displays to a subject's self-motion. VR ‘CAVE’ image from: https://commons.wikimedia.org/w/index.php?curid=868395.

Real environments	Large display environments	Head-mounted displays
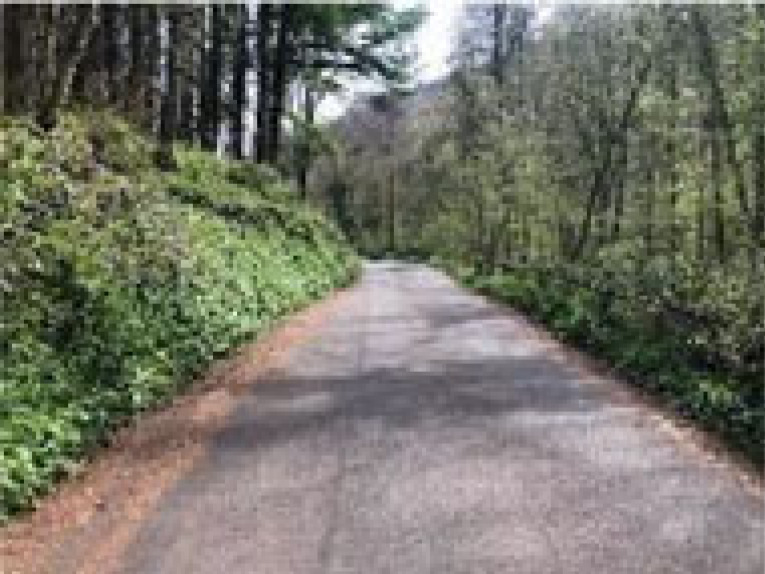	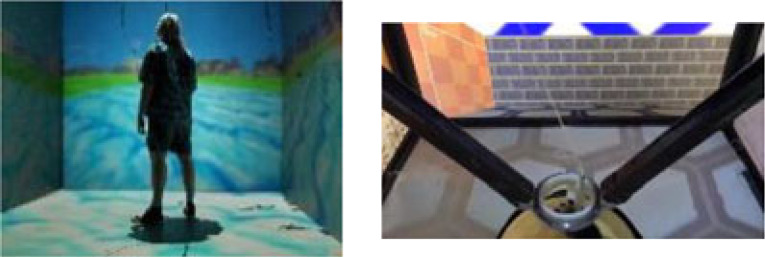	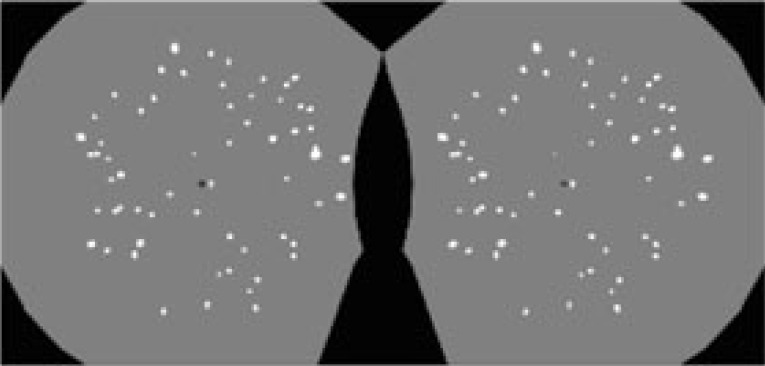
A photograph of a woodland. — Natural visual inputs. — World and eye cameras can be used to reconstruct observed visual scene [9,103].— Lenses [106] and prisms [107] can be used to manipulate visual inputs.	*Left*: a VR ‘CAVE’. *Right*: two-dimensional rodent VR [102]. — Flexible presentation of visual inputs. — Curved or multiple display devices can achieve large visual field coverage. — Virtual [95,108,109] / augmented [108,110,111] reality.	Left and right eye views of a head-mounted display. — Flexible stereoscopic presentations of visual inputs. — Virtual [104] / augmented reality [105].
Coupling with movement		
— Natural free movement. — Passive motion platforms and treadmills can be incorporated [112].	— One-dimensional/two-dimensional treadmills [49,72,102]. — Passive motion platforms [113,114]. — Free movement interaction is possible with motion tracking cameras (e.g. ‘VR CAVEs’) [115].	— Natural free movement using built-in tracking of rotational and translational head movements. — One-dimensional/two-dimensional treadmills [104]. — Passive motion platforms.

**Table 2.** An overview of the main features of different experimental methods used to investigate visual processing and perception in moving subjects.

Type of movement	Active self-motion?	Proprioceptive cues?	Vestibular cues?
Free movement	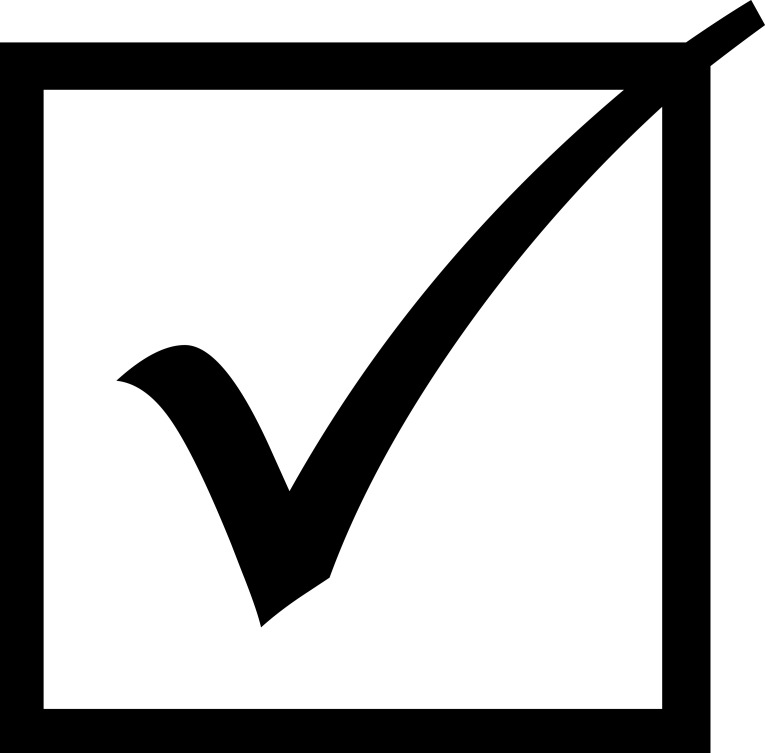	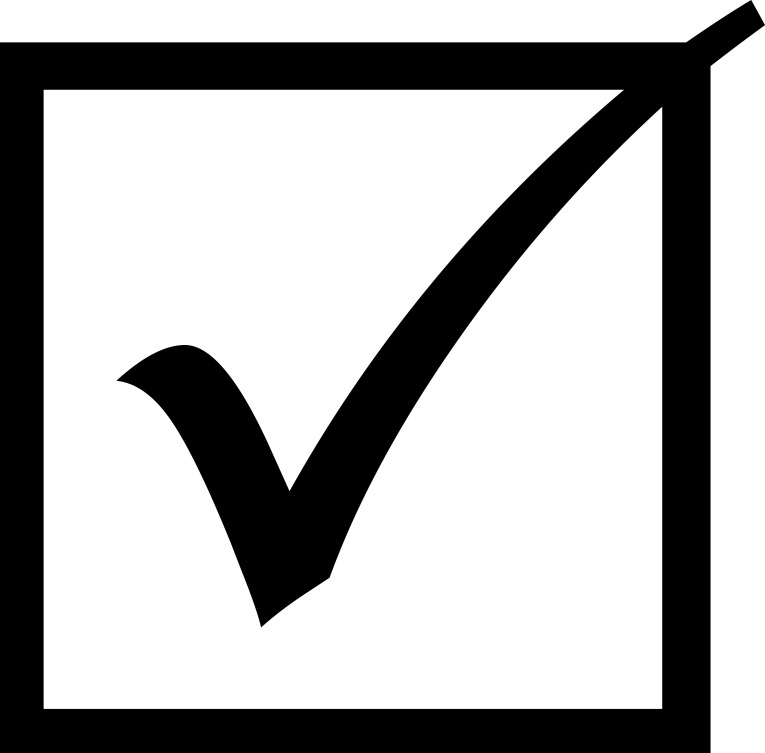	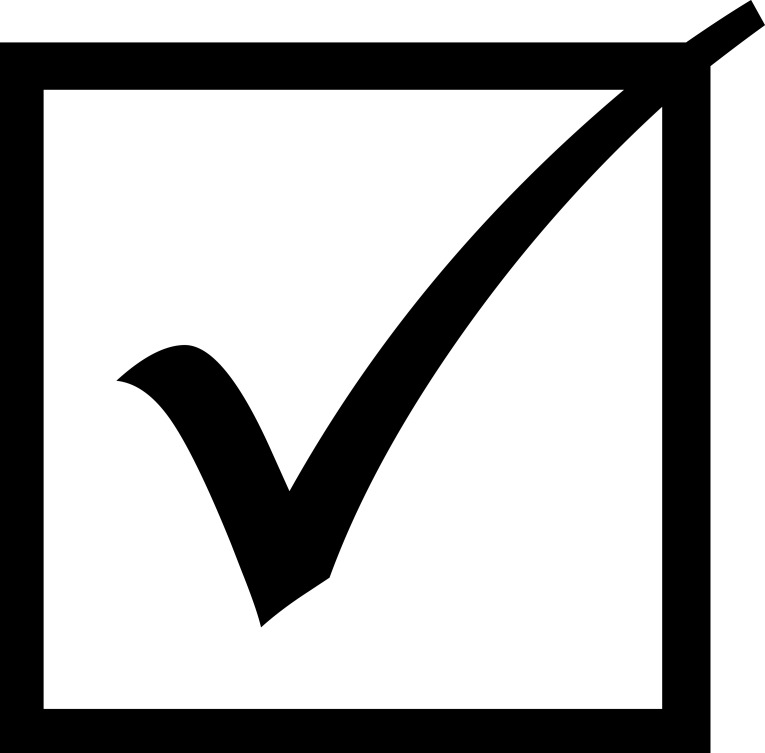
Treadmills	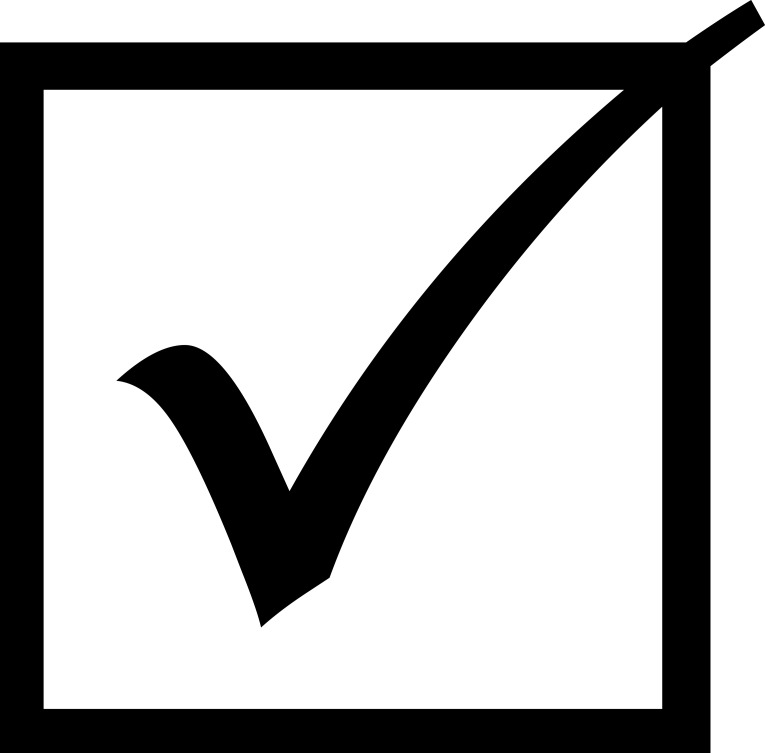	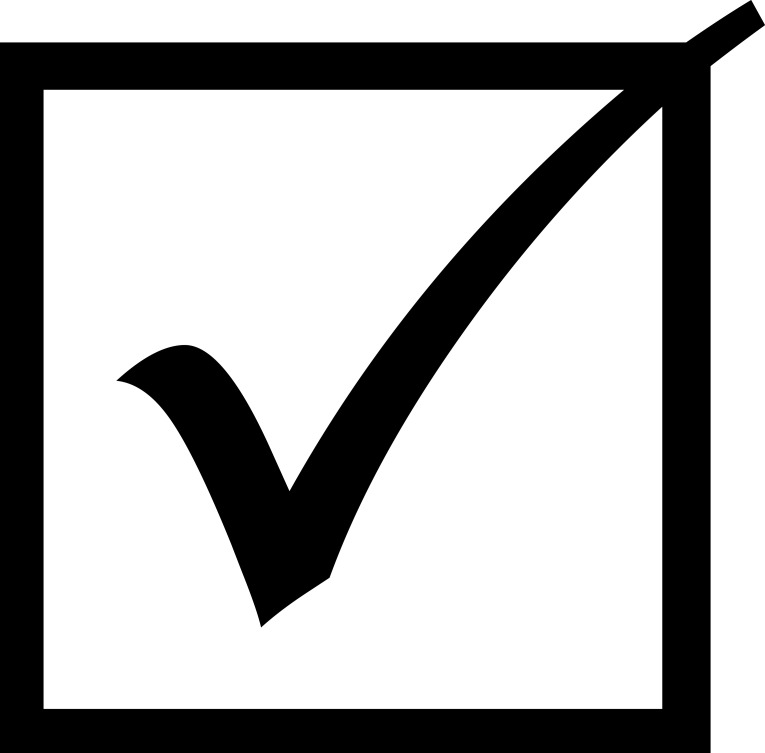	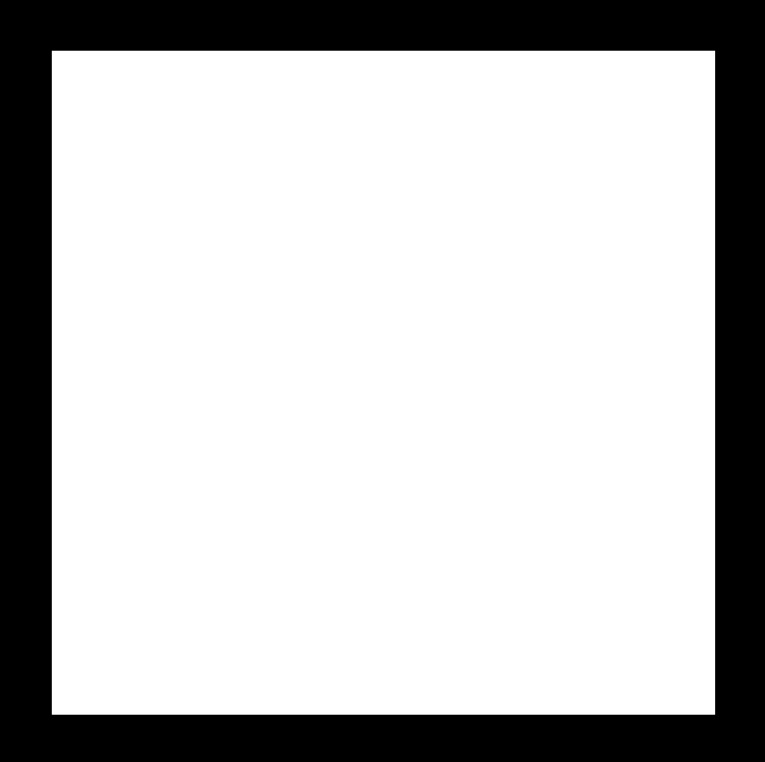
Motion platforms	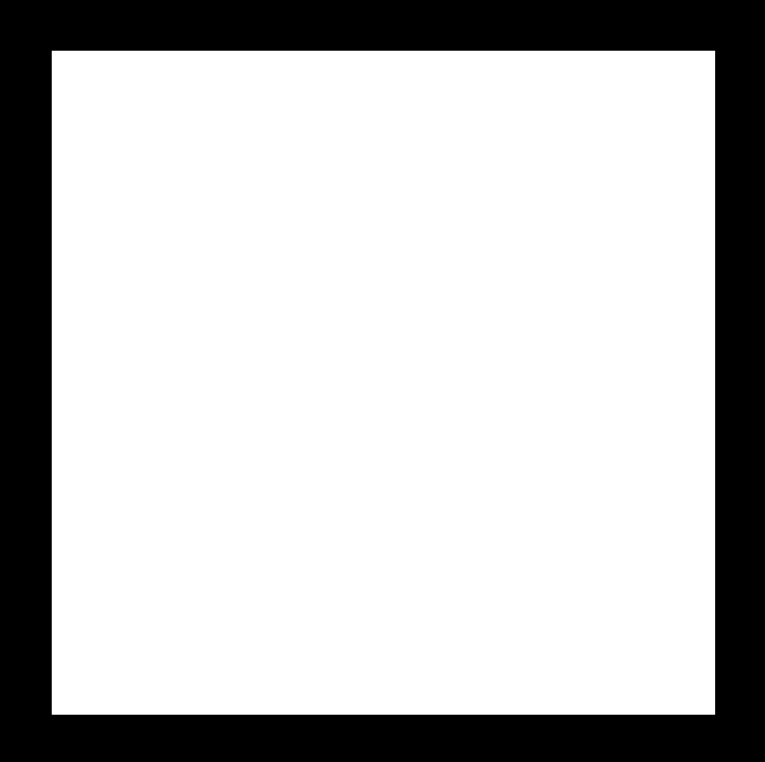	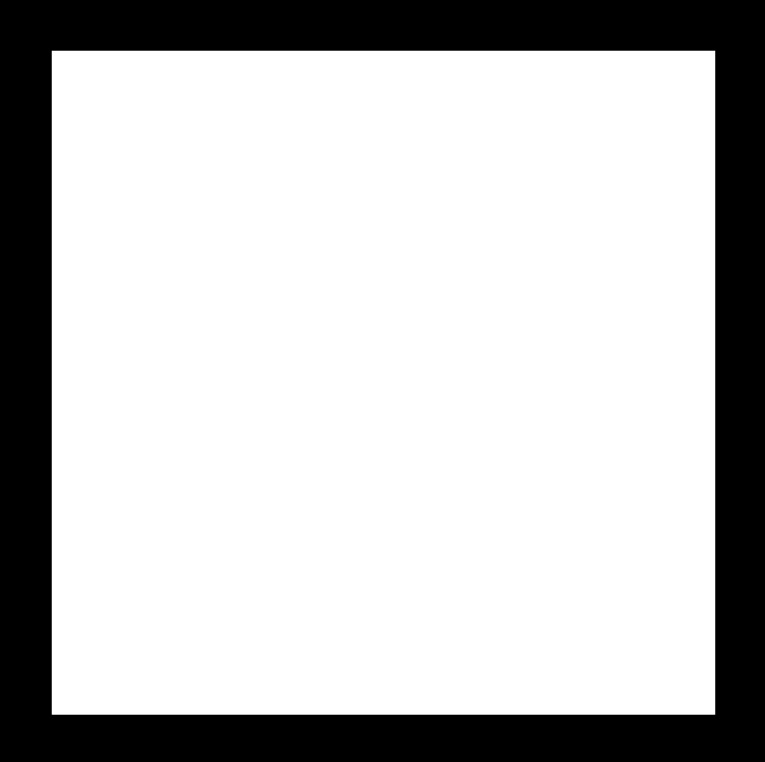	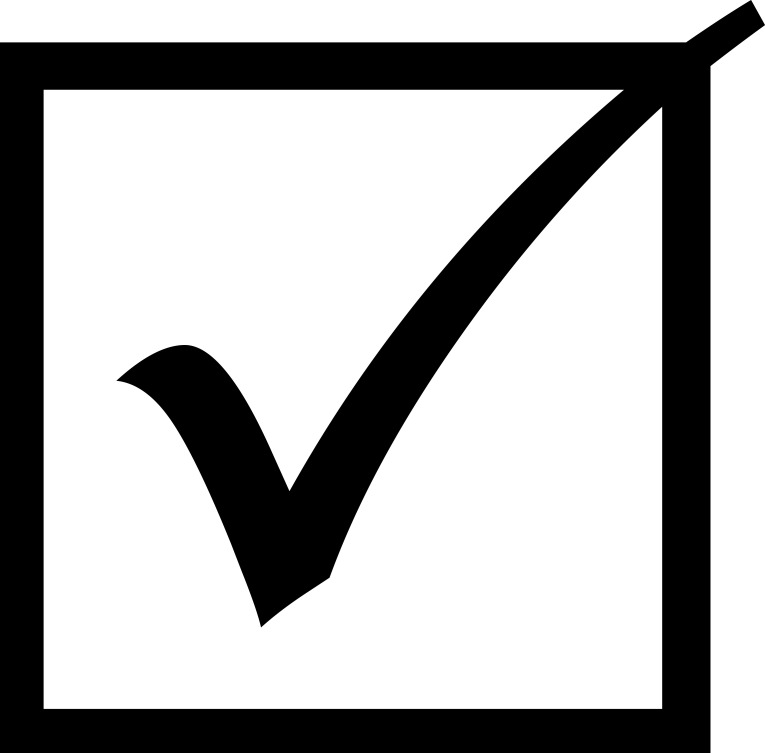

## 2. Key findings

In the following sections, we highlight key findings regarding how the perception and neural encoding of optic flow are affected by movement related to locomotion. We begin each section with a description of key observations made using psychophysics in human subjects. We then discuss physiological and perceptual measurements in animal models that may provide mechanistic explanations for the observed effects, focusing on findings we feel are most relevant to perceptual phenomena.

### (a) Walking slows perception of optic flow speed

**Figure 2.**
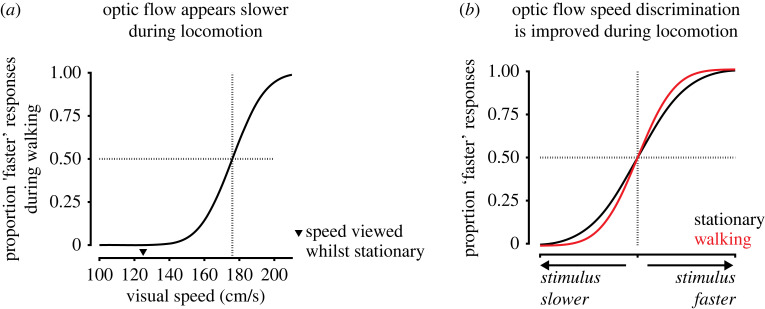
Changes in optic flow speed perception during locomotion. (*a*) While walking, subjects perceive faster optic flow speeds to match optic flow speeds viewed when stationary, indicating that optic flow appears slower. This is illustrated by the point of subjective equality (indicated by dashed lines) being shifted to a faster visual speed compared to a reference speed (indicated by black triangle) viewed when stationary (illustration of results from [11]). (*b*) During walking subjects exhibit increased sensitivity to optic flow speeds faster than a threshold speed that approximately matches average walking speed (approx. 125 cm s^−1^ in [11]). As a result, psychometric curves for optic flow speed discrimination are slightly steeper when subjects are walking, indicating improved discrimination of optic flow speeds (illustration of results from [11]).

A striking phenomenon of human locomotion is that optic flow appears slower while walking [11,104,116–118]. Specifically, walking subjects choose faster optic flow speeds to match optic flow speeds viewed while stationary (figure 2*a*; [11,104]). This suggests that locomotion has a subtractive effect on the perception of optic flow speed. This subtractive effect is largely specific to walking as there is a reduced effect when self-motion is related to cycling or arm-cycling and no observable effect for an arbitrary periodic action (finger tapping; [119]). The subtractive effect can be present during treadmill walking, suggesting a role for proprioceptive and/or efference copy signals [104]. It is also observed during passive translation in a wheelchair [104], suggesting a role for vestibular signals. Normal walking approximately sums the effects of treadmill walking and passive translation in a wheelchair, indicating that nonvisual self-motion signals additively contribute to the magnitude of the effect [104]. Importantly, the effect also scales with walking speed, indicating that it goes beyond a binary behavioural state-dependent effect, and instead depends on continuous self-motion signals correlated to walking speed [104,119,120]. The effect also depends on the congruence of visual motion to self-motion [104,116] and is larger for faster visual speeds [117], demonstrating that it depends jointly on visual and nonvisual self-motion signals.

Is there a neural mechanism for optic flow to be perceived as being slower during locomotion? It is now well established that the encoding of visual speed by neurons throughout the mouse visual system is dependent on concurrent self-motion signals [71,77,80,81,95]. Of particular relevance, it has been reported that neurons in mouse V1 and higher visual areas AL and PM prefer faster visual speeds during locomotion [81]. If neural tuning changes between behavioural states, a downstream area trained to estimate visual speed using neural activity in one behavioural state could make errors estimating visual speed from neural activity occurring in another state. Therefore, systematic changes to tuning preferences during locomotion might result in biased predictions. Specifically, if a decoding area generates a model of visual speed tuning based on when the animal is stationary and subsequently attempts to decode visual speed when the animal is locomoting, it may underestimate visual speed since the neurons it is decoding from now prefer faster visual speeds. Thus, changes in visual speed tuning preferences between behavioural states are a potential neural mechanism underlying changes in the perceived speed of optic flow. However, it is not yet known whether visual speed tuning preferences similarly change between stationary and locomoting states in primates, and comparisons of mouse visual speed perception between stationary and locomoting states are not yet available. Future experiments comparing neural tuning for visual speed between stationary and locomoting states in head-fixed marmosets [101], alongside experiments investigating changes in perceptual estimation of visual speed in mice, would provide the means to test this.

### (b) Walking improves optic flow speed discrimination

What is the function of the slowing down of perceived optic flow speed during locomotion? One hypothesis is that it enables increased sensitivity to optic flow speed [11]. This hypothesis is based on the framework for a perceptual coordinate system for correlated variables proposed by Barlow [121,122]. The framework is based on an assumption that a subject can discriminate a fixed number of divisions of perceived optic flow speeds. Therefore, when walking reduces the perceived speed of optic flow it also reduces the range of perceived speeds that need to be encoded by a subject's visual system. This then enables the discrimination of finer differences of speed [11,104,121,122]. In agreement with this hypothesis, locomotion can improve the discrimination of optic flow speed (figure 2*b*; [11]). A similar result has been demonstrated using an adaptation paradigm [123]. Subjects who viewed an initial adapting stimulus that was moving were subsequently biased to perceive a test stimulus as moving with a slower speed. Interestingly, the adapting stimulus also improved subjects’ discrimination of the speed of the test stimulus, with improvements in discrimination proportional to the magnitude of perceptual bias. Thus, it may be a general principle that adaptation can increase sensitivity to visual motion at the cost of biasing perception, prioritizing sensitivity over accuracy [124].

We recently showed that the visual speed of moving dot fields can be better decoded from neural activity in locomoting mice [71], raising the possibility that perceptual sensitivity for visual speed is also improved in mice during locomotion, similar to humans [11]. We also found that visual speed could be decoded earlier following stimulus onset in locomoting mice [71], potentially reflecting an adaptive neural mechanism enabling mice to respond more rapidly to changing visual motion inputs. Improvements in the neural encoding of visual speed during locomotion vary between visual areas and are strongest in V1 and medial higher visual areas AM and PM [71]. Interestingly, areas AM and PM are biased to respond to the peripheral visual field [62] where changes in optic flow during locomotion are largest and may be best processed [125]. Mouse higher visual areas AM and PM may therefore be specialized for the neural encoding of optic flow during locomotion. Perceptual experiments will be required to determine whether locomotion also improves perceptual sensitivity for visual speed in mice, as well as providing a means to investigate the neural mechanisms underlying any changes in perception.

### (c) Self-motion alters flow parsing and heading perception

**Figure 3.**
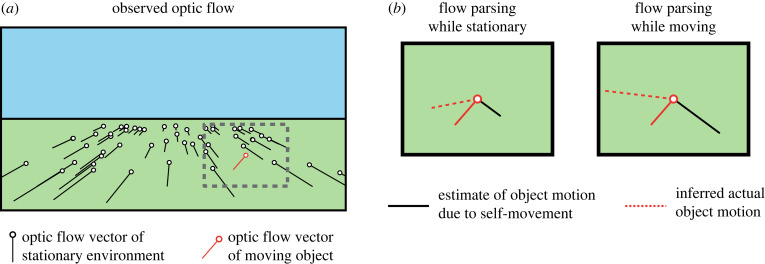
Changes in flow parsing during self-motion. (*a*) Forwards translation over a simple ground plane produces a pattern of expanding optic flow (black lines). Observed visual motion of an object within the environment depends on a combination of simulated self-motion and object motion. (*b*) Shown is a zoomed-in view of the dashed grey box in (*a*). The flow parsing hypothesis posits that observers infer actual object motion (dashed red lines) by a vector subtraction of the estimated visual object motion due to self-motion (solid black lines) from observed object motion (solid red lines). The estimate of visual motion due to self-motion is larger in magnitude when an observer is moving (*Right panel*) compared to while stationary (*Left panel*), resulting in changes to inferred actual object motion (illustration of results from [129] based on schematic from [130]).

The subtractive effect of locomotion on the perceived speed of optic flow may also reflect perceptual stabilization of the visual environment [126], enabling the detection of motion within the environment by parsing visual motion caused by external motion from optic flow caused by self-motion. Consistent with this hypothesis, when visual object motion is presented simultaneously with optic flow simulating self-motion, humans and non-human primates can infer object motion by subtracting a visual estimate of self-motion based on the global optic flow pattern (‘flow parsing’; [25,127]). While flow parsing has primarily been investigated as a visual process [128], the subtractive effect of self-motion on optic flow speed perception indicates that nonvisual signals may also contribute. Moreover, congruent vestibular stimulation by passive translation promotes the perception of pattern motion when viewing bistable plaid stimuli [113], indicating that, when available, nonvisual self-motion signals contribute to the perception of global motion patterns such as optic flow. Indeed, in primates (both human and non-human) the inclusion of congruent vestibular signals via passive translation increases the compensation for visually simulated self-motion when judging object motion trajectories (figure 3; [129,131]). Furthermore, by shifting visual heading relative to walking direction within a virtual environment, it has been demonstrated that humans can use a combination of visual and nonvisual cues for self-motion to plan future interactions with objects [130,132]. Thus, when nonvisual self-motion signals are available they can contribute to the perceptual parsing of visual motion due to object motion from optic flow generated by self-motion.

**Figure 4.**
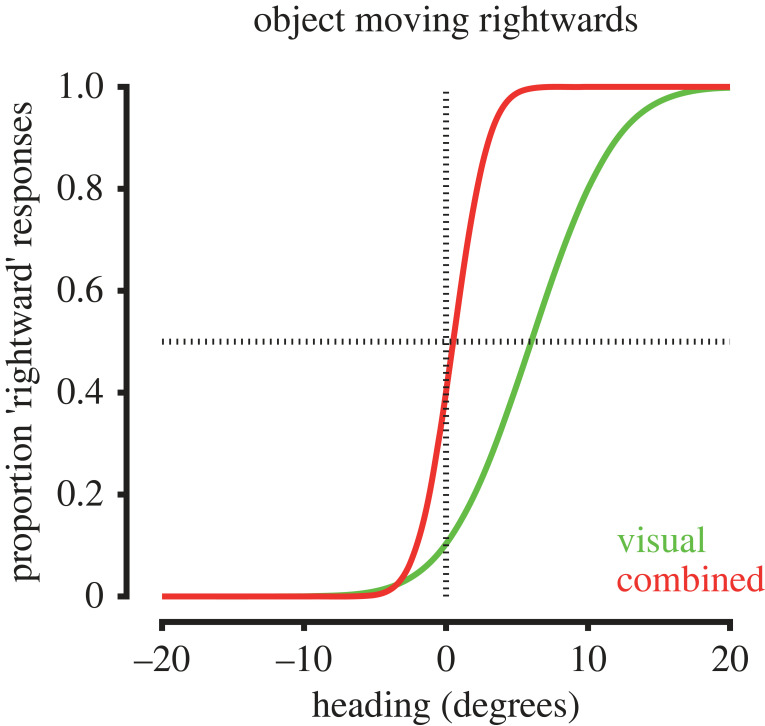
Changes in estimates of heading in the presence of object motion during self-motion. The presence of moving objects can bias estimates of heading from optic flow. For example, the presence of a rightward moving object can bias estimates of visual heading to the left (green trace). The addition of congruent vestibular signals reduces these biases resulting in more accurate estimates of heading (red trace). Illustration of results from [133].

Self-motion signals can also improve the accuracy of heading direction perception from optic flow in the presence of interfering object motion. While humans and non-human primates can accurately judge self-motion heading from optic flow in stationary environments, the presence of object motion can bias estimates of heading (figure 4; [134,135]), particularly when optic flow is unreliable [133]. Thus, perceptual estimation of heading direction on the basis of visual cues alone is prone to errors in visually ambiguous settings. However, the inclusion of congruent vestibular stimulation via passive self-motion significantly reduces object motion-induced biases in heading perception (figure 4; [133]). Congruent vestibular inputs can therefore improve the accuracy of heading perception when visual object motion is present within the environment.

The neural mechanisms underlying flow parsing in primates are not well established. Candidate areas which may play a role in the primate visual system are the Middle Temporal (MT) area and MSTd [129], which are reciprocally connected [136]. Neurons in MSTd often have receptive fields that cover the majority of the visual field [137] and can encode heading direction from mixtures of visual and vestibular inputs [114,138]. Neurons in area MT have smaller receptive fields, are commonly tuned for visual motion direction and speed and exhibit anatomical clustering based on motion direction and speed preferences [139,140], making them suitable for the encoding of local object motion. As such, heading estimates based on activity in MSTd may be fed back to influence the representation of object motion in area MT (and vice versa). Neurons in MSTd that selectively respond to incongruent combinations of visual and vestibular motion direction may also play a role in assigning visual motion due to object motion during self-motion [141].

In the mouse, the higher-order visual thalamic Lateral Posterior (LP) nucleus (analogous to the primate pulvinar) contains neurons tuned for negative correlations between visual speed and running speed [77,80]. Projections from LP also appear to be the major driver of joint tuning for visual speed and running speed in higher visual cortical area AL, which also contains neurons that primarily encode negative correlations between visual speed and running speed [80]. These neurons are reminiscent of cells found in primate MSTd that encode incongruent combinations of visual and vestibular motion direction [141] and suggest a possible role for LP and AL in detecting object motion during self-motion. Interestingly, anatomical subregions of LP with distinct functional connectivity to other visual areas appear specialized for the encoding of either full-field or object motion [142], further indicating that LP may play a central role in flow parsing within the mouse visual system. The emergence of a flow parsing task for non-human primates [129] and the development of appropriate behavioural tasks for mice should provide more opportunities to investigate the neural mechanisms underlying the perceptual parsing of visual motion due to object motion from self-motion generated optic flow.

### (d) Visual and nonvisual signals are integrated for perception of self-motion

**Figure 5.**
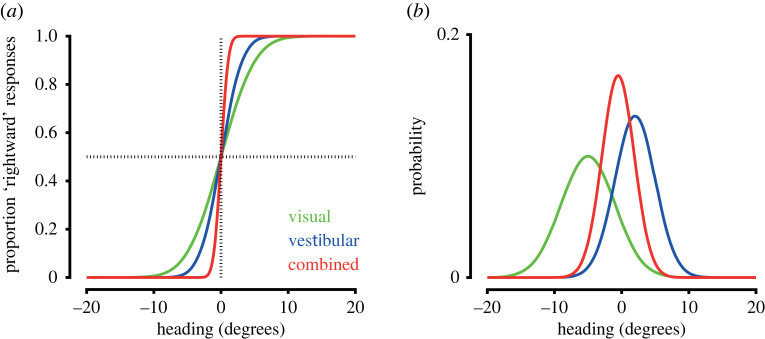
Integration of visual and nonvisual self-motion signals. (*a*) Perceptual estimates of heading are more precise when both visual and nonvisual self-motion signals are present compared to unisensory conditions, resulting in steeper psychometric curves (red trace versus green and blue traces). (Illustration of results from [133]. (*b*) The integration of visual and nonvisual signals for estimating self-motion is largely consistent with Bayesian cue integration. Illustrated here for heading direction, the mean of the multisensory estimate (red trace) is a weighted sum of unisensory estimates from visual (green trace) and vestibular (blue trace) cues, with cue weights proportional to their reliability. The uncertainty of the multisensory estimate is reduced compared to unisensory estimates.

The perception of self-motion is a multisensory experience, combining visual, proprioceptive and vestibular signals with a range of other cues. Humans and non-human primates can integrate visual and nonvisual cues to estimate heading direction [143,144] and distance travelled [145–148], which can in turn increase perceptual precision (figure 5*a*; [133,149–151]). Integration of visual and nonvisual self-motion signals is largely consistent with Bayesian cue integration, whereby perceptual weights for each cue are proportional to their reliability, albeit with a tendency to overweight body-based vestibular (figure 5*b*; [149–151]) and proprioceptive cues [146–148]. Notably, inclusion of stereoscopic visual information results in more optimal cue integration in humans [152], indicating that the richness of sensory information available may be important for how cues are integrated. More generally this suggests that the integrative process is flexible and context-dependent.

In primates, multisensory perception of heading direction has been strongly linked to activity of neurons in MSTd which respond to combinations of optic flow and vestibular input using a reliability-dependent weighted sum [153,154]. In addition, causally manipulating MSTd activity can alter behavioural reports of heading perception [155,156], indicating that it plays an important role in primate multisensory heading perception.

Recent experimental findings demonstrate that mice can also combine vestibular and visual cues to improve perceptual discrimination of angular velocity [157], as has been demonstrated in humans [158]. Mouse perceptual performance could be accounted for by linear decoding of neurons tuned to both visual and vestibular cues in the retrosplenial cortex [157], an area that may be important for integrating visual, vestibular and motor signals associated with self-motion in rodents [17] and also conveys vestibular information and influences turning-related signals in mouse V1 [103,159–161]. Mice also integrate visual and nonvisual signals to estimate distance travelled [162]. Mice trained to lick for reward at a given visual location within a virtual corridor exhibited biased licking responses when the visual gain of the environment was altered [162], indicating that they combined visual and nonvisual signals to estimate distance travelled. Simultaneously measured spatial position tuning of neurons in primary visual cortex and hippocampal CA1 shifted during gain changes, with the resulting decoded spatial position from these neurons corresponding well with the animals' behavioural performance [162,163]. Additionally, a range of mouse visual areas contain neurons tuned to combinations of visual speed and locomotion speed [77,80,95] which may play a role in the integration of visual and nonvisual self-motion signals for perceptual inference of self-motion speed. Thus, in both primates and mice, perception of self-motion is based on integration of optic flow with nonvisual signals.

### (e) Visual and nonvisual self-motion signals are dynamically calibrated

Multisensory integration leverages the relationships between different senses to enable more precise perceptual judgements [133,164]. However, the relationship between the visual and nonvisual signals associated with locomotion is contingent upon environmental context. For example, the visual speed of a textured surface is inversely proportional to its viewing distance. As such, a dynamic, context-dependent calibration process is necessary to maintain a valid model of the relationship between nonvisual locomotion signals and optic flow (multisensory cue calibration). Such dynamic calibration of visual and nonvisual signals has been observed in both humans and non-human primates using a number of experimental paradigms [112,132,165–170]. Many of these studies involved exposing subjects to a recalibrating context, with the after-effects of a potential recalibration assessed in a short time period following exposure. In one set of experiments, the recalibrating context was walking on a treadmill [166]. After spending approximately 10 min walking on a treadmill, subjects reported an accelerated perception of self-motion speed while walking normally in stationary surroundings. This effect was quantified by the time taken to walk a 5 m lap following treadmill exposure—despite subjects being instructed to walk at a constant, pre-specified speed, they gradually sped up over a duration of 2–3 min. This result can be interpreted as a series of recalibrations between walking speed and optic flow. During treadmill walking subjects recalibrated a slower optic flow speed to be associated with a given walking speed due to the lack of normal optic flow on a treadmill. When subjects were subsequently re-exposed to the normal contingency between optic flow and walking, the addition of normal optic flow then produced an accelerated sense of self-motion. As subjects then recalibrated to the normal contingency between optic flow and walking, the effect of treadmill walking wore off and subjects increased their walking speed to maintain a perception of constant perceived self-motion speed.

In another set of experiments subjects walked on a treadmill while being pulled by a tractor moving either faster or slower than treadmill speed so as to alter the gain of optic flow associated with locomotion and induce visuomotor recalibration [112]. Afterward, subjects were shown targets at a distance and, subsequently, asked to walk to them while blindfolded. Subjects who had been pulled by the tractor slower than their walking speed (low visual gain) overestimated distance to the targets, suggesting that they had calibrated a longer walking distance to be necessary to travel a set visual distance. By contrast, subjects pulled faster than their walking speed (high visual gain) underestimated distance to targets. Interestingly, a related study found that prolonged walking on a rotating circular disc in a visually stationary room caused subjects to subsequently walk in a circular trajectory when trying to walk in place while blindfolded [165], indicating that subjects had recalibrated a circular walking trajectory to be required to remain visually stationary. Thus, humans dynamically recalibrate nonvisual locomotion signals with optic flow, even when the contingency between them is unusual.

A common perceptual bias in virtual reality environments is the underestimation of distances. However, experience of closed-loop feedback can significantly improve these perceptual judgements [171–174], indicating that a calibration process between self-motion and corresponding optic flow can reduce naively occurring biases in virtual environments. The mechanisms underlying this recalibration remain poorly understood, although experiments manipulating the availability of specific visual cues may provide useful insights [34].

It has been proposed that multisensory cue calibration consists of two distinct processes—‘unsupervised’ and ‘supervised’ calibration [175,176]. Unsupervised cue calibration acts on cues that signal discrepant information by shifting perceptual estimates based on each cue toward each other. Unsupervised cue calibration, therefore, aims to achieve ‘internal consistency’ and does not depend on external feedback. By contrast, supervised cue calibration uses external feedback to shift perceptual estimates based on each cue with the aim of achieving accurate perception of the environment.

**Figure 6.**
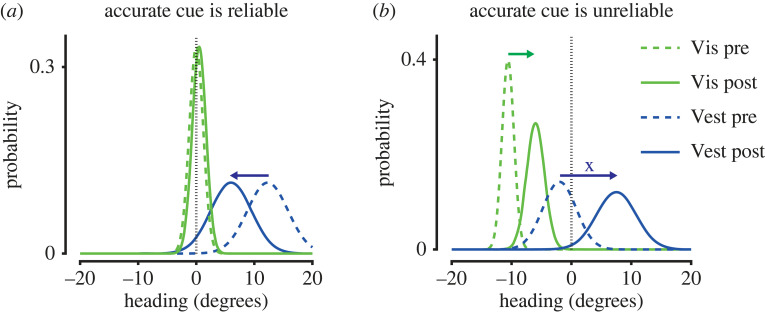
Supervised calibration of visual (Vis) and nonvisual (Vest) self-motion signals. Supervised cue calibration depends on both the accuracy and reliability of cues. This is illustrated here for heading direction. The actual heading is indicated by the vertical dashed line and the reliability of cues is indicated by the relative variance of their distributions. (*a*) When the accurate cue is the most reliable, the inaccurate cue is calibrated alone. (*b*) When the accurate cue is the least reliable, both cues are calibrated together. (Illustration of results from [176]).

Multisensory cue calibration underlying the perception of heading direction has been investigated using an adaptation paradigm [175,176]. In a series of experiments, unisensory heading direction perception of humans and non-human primates was tested following adaptation to a visual-vestibular contingency whereby visual heading was 10° offset to heading signalled by vestibular stimulation during passive self-motion [175,176]. By contrast to multisensory integration, unsupervised cue calibration was independent of the relative reliability of cues (controlled by the motion coherence of the optic flow stimulus). Instead, vestibular adaptation was approximately twice as strong as visual adaptation, as assessed by reports of heading direction reported in unisensory trials following adaptation [175]. However, during supervised calibration, which was controlled using reward as external feedback for cue accuracy, calibration depended on both cue reliability and accuracy. When the less reliable cue was also inaccurate it was calibrated alone (figure 6*a*), but when the more reliable cue was inaccurate both cues were calibrated together as a combined percept (figure 6*b*). As a result, the reliable, inaccurate cue became more accurate, however, the less reliable, initially accurate cue became less accurate [176]. A recent study investigating the neural correlates of supervised multisensory calibration found that neurons in the Ventral Intraparietal (VIP) area, but not MSTd, exhibited shifts in tuning for vestibular and visual heading following adaptation that were correlated with behavioural performance in the task [177], in line with increased choice-related activity in VIP [178].

The neural mechanisms underlying cue calibration of visual and nonvisual self-motion signals have not been explicitly investigated in the mouse. A subset of V1 neurons has been identified that may play a role in detecting discrepancies between optic flow and nonvisual self-motion signals, as would occur during unsupervised calibration [109]. These neurons selectively respond to halts or perturbations of optic flow during locomotion [109,179,180]. While these responses are partly influenced by inputs from secondary motor and anterior cingulate cortex [181,182], the precise mechanisms driving responses to optic flow perturbations are debated [180]. Regardless of their mechanism, the responses of such neurons may be suited to trigger recalibration of optic flow and nonvisual self-motion signals by signalling when these signals disagree. Experiments combining perception and neurophysiology, alongside modelling approaches, would provide a means of investigating self-motion related multisensory cue calibration in mice and determine whether similar principles apply to those observed in primates.

## 3. Some considerations

### (a) Dissociating the effects of self-motion from changes in behavioural state

A key challenge for future research is dissociating effects of self-motion from co-occurring changes in behavioural and brain state. The mouse, in particular, has provided a number of insights into the effects of behavioural state on visual processing and perception [13,15,96,183,184]. For example, orofacial movements including whisking strongly correlate with locomotion and can drive large changes in neural activity [98,184,185], which if not carefully accounted for can confound inferences about neural activity underlying perception and behaviour [186]. Comparing the effects of different types of active and passive self-motion [104,187], alongside manipulation of visual inputs and their relationship to self-motion [82,104,187], can be used to carefully dissect and dissociate the various factors that may contribute to the influence of self-motion on visual processing and perception. A further consideration is that both mice and humans make distinct sets of eye movements during unrestrained active self-motion in order to stabilize visual flow [9,18,103,188], indicating that locomotion is associated with distinct perceptual strategies for sampling the visual scene. Moving forward, careful quantification of brain and behavioural state information [184,186,189–191] will be essential to dissociate the various factors that co-occur with locomotion.

### (b) Conscious awareness

To what extent does the integration of visual and nonvisual cues for self-motion occur without conscious awareness? Humans automatically adjust their locomotion speed to changes in optic flow patterns [21,192–194] and these adjustments are present in subjects with cortical blindsight [118], suggesting that visual control of locomotion can occur without explicit conscious awareness in humans. Changes in locomotion speed also bias human subjects' ability to discriminate between visual speeds in a 2-interval forced choice task [117], indicating that there is a degree of mandatory perceptual fusion of optic flow with nonvisual self-motion signals that prevents conscious access to isolated optic flow during locomotion. Evidence for the mandatory fusion of vestibular and visual cues has also been observed for the perception of heading direction [158], suggesting that this may be a general phenomenon in the multisensory perception of self-motion.

## 4. Conclusion and future directions

In this review, we brought together findings from distinct fields of research. While the influence of self-motion on optic flow perception has been most-studied in humans and non-human primates, the effects of locomotion on neural activity are best characterized in mice. We believe each of these species affords unique insights into the influence of self-motion on optic flow processing and perception, and ultimately that a multi-species approach, therefore, provides the best way forwards. To this end, we hope this review provides a useful starting point by highlighting existing cross-over in these areas of research as well as the distinct experimental opportunities afforded by each of these species.

Experimental approaches to investigate vision during movement have had various constraints in available methodologies for each species. For example, non-human primate studies have generally investigated the influence of vestibular signals on optic flow processing and perception during passive movement using motion platforms. However, developing technologies are expanding experimental opportunities (see [195] for a useful review of recording methodologies available in moving subjects). For example, recordings of head-restrained marmosets free to locomote on a treadmill [101] should enable the investigation of optic flow processing and perception during active locomotion in a non-human primate and therefore enable more direct comparisons with findings from mice using similar experimental assays.

The recent development of lightweight eye tracking cameras for mice [9,103,188,196–198] enables simultaneous monitoring of head and eye movements during free movement, making it possible to reconstruct on a moment-by-moment basis the visual scene as viewed by a subject. Continued development of this technology alongside appropriate analysis methods will enable experimental investigation of visual encoding and perception in unrestrained, naturally behaving subjects, for example during prey capture [8,9] or predator avoidance [10]. This can be further enhanced by the ability to present interactive augmented reality environments to animals [110,111].

In humans, wireless head-mounted displays (HMDs) allow for precise control of binocular visual stimulation to a subject during active unrestrained movement. The continued development of this technology should allow for more comfortable, realistic and immersive visual experiences. Moreover, a number of groups have begun to combine head-mounted displays with non-invasive recording methods such as electroencephalography [199], and functional near-infrared spectroscopy [200], enabling simultaneous recording of neural activity during visual behavioural tasks.

As the availability of experimental technologies increases, so does the capacity for analogous experiments across species. This in turn allows for more direct comparisons of the effects of self-motion on optic flow processing and perception. Indeed, advances in training protocols mean that mice can now be routinely trained to perform a range of visual psychophysics tasks [100,157,159,201–204] similar to those successfully used to investigate visual perception in humans and non-human primates. Using approaches across animal species and with different brain recording techniques, we are now in a strong position to investigate the influence of self-motion on optic flow processing and perception. These approaches will be essential to understand how animals successfully interact with dynamic environments and should, moreover, provide insights into principles of sensorimotor coding in mammalian species.

## Data Availability

This article has no additional data.
